# Childhood Trauma and Its Relation to Chronic Depression in Adulthood

**DOI:** 10.1155/2015/650804

**Published:** 2015-11-29

**Authors:** Alexa Negele, Johannes Kaufhold, Lisa Kallenbach, Marianne Leuzinger-Bohleber

**Affiliations:** ^1^Sigmund-Freud-Institute, 60323 Frankfurt, Germany; ^2^University of Kassel, Psychoanalytical Psychology, 34127 Kassel, Germany

## Abstract

There is a large consensus indicating that childhood trauma is significantly involved in the development of depression. The aim of this study was to examine the prevalence of retrospectively recalled childhood trauma in chronically depressed patients and to investigate a more specific relationship between trauma type and depression. We further asked for the influence of multiple experiences of childhood trauma on the vulnerability to a chronic course of depression in adulthood. 349 chronically depressed patients of the German LAC Depression Study completed the Childhood Trauma Questionnaire, a self-report measure of traumatic experiences in childhood. 75.6% of the chronically depressed patients reported clinically significant histories of childhood trauma. 37% of the chronically depressed patients reported multiple childhood traumatization. Experiences of multiple trauma also led to significantly more severe depressive symptoms. Stepwise multiple regression analysis suggested that childhood emotional abuse and sexual abuse were significantly associated with a higher symptom severity in chronically depressed adults. Yet, expanding the regression model for multiple exposures showed that multiplicity was the only remaining significant predictor for symptom severity in chronically depressed patients. Clinical implications suggest a precise assessment of childhood trauma in chronically depressed patients with a focus on emotional abuse, sexual abuse, and multiple exposures to childhood trauma. This trial is registered with registration number ISRCTN91956346.

## 1. Introduction

Among the leading causes of the global burden of disease depression currently ranks third place worldwide and first place in middle- and high-income countries [1, p. 43]. Wittchen et al. [2] even reported depression to be “by far the most burdensome disorder of all diseases in the EU” (p. 669). It is predicted that by 2020 depression will have jumped to second place [3]. Depression has a high risk of recidivism: 50% after the first, 70% after the second, and 90% after the third episode [4]; 50% of patients relapse after any form of short-term psychotherapy [5]. 20 to 30% of patients with major depression do not respond to antidepressant medication and one-third of those who initially responded relapse within a year [6]. Depressed patients form a heterogeneous group showing quite different pathogeneses. Traumatic experiences in childhood can be found in most multifactor models on etiopathogenesis as a psychosocial aspect of depression (e.g., [7–10]). Epigenetic studies further substantiate the finding that a genetic vulnerability will only lead to depression if the individual experienced simultaneous early traumatization. Caspi et al. [11] showed that early separation trauma triggers the 5-HTTLPR allele in turn regulating relevant neurotransmitters hence evoking depression. Childhood trauma may be one decisive source of heterogeneity that may also depend on trauma type [10].

Trauma can be understood as a “relational” term, a concept that connects an outer occurrence with its specific consequences for an inner psychic reality [12, 13]. Referring to Cooper [14] psychic trauma is any psychological event that abruptly overwhelms the capacity to* “provide a minimal sense of safety and integrative intactness, resulting in overwhelming anxiety or helplessness, or the threat of it, and producing an enduring change in the psychic organization”* (p. 44). With single trauma versus multiple trauma the accumulation of traumatic experiences can be differentiated. In multiple trauma different traumatic events or situations can either simultaneously, sequentially [15], complexly [16, 17], or cumulatively [18] be effective and thereby manifold their impacts [12, 19–21]. Terr [22] differentiated between type I and type II trauma. Type I trauma conceptualized as “one sudden blow” (p. 13) following from an “unanticipated single event” (p. 14). Type II trauma refers to “longstanding or repeated exposure to extreme external events” (p. 15).

Clinical and survey studies show in general a significantly higher prevalence of childhood trauma in mental disorders (e.g., [12, 23, 24]) emphasizing a nonspecificity of traumatic experiences as risk factors for the vulnerability to different forms of psychopathology. Nonetheless, the relationship of childhood trauma and an increased risk of depression in adulthood has been confirmed in several cross-sectional (e.g., [25, 26]) and longitudinal (e.g., [27, 28]) studies. Most studies rely on either community based surveys (e.g., [26, 27, 29–31]) or psychiatric outpatient samples with heterogeneous (e.g., [24, 32]) or homogeneous (e.g., [33]) psychopathology and also ask for a possible relative specificity of trauma types and diagnoses.

For example, several studies (e.g., [10, 34, 35]) documented the association of sexual abuse in childhood and depression in adulthood. Molnar et al. [26] showed that among men and women suffering from sexual abuse the risk of developing a depression was 1.8 times higher. Others [34] even discuss four times higher risk. Fergusson and Mullen [34] reported in their community survey that 60% of the women with exposure to childhood sexual abuse fulfilled the criteria of a chronic major depression. Furthermore, empirical findings showed that emotional abuse (e.g., [23, 30–32]) and emotional neglect (e.g., [10, 27, 31, 33]) increase the vulnerability to develop a major depression in adulthood. The more chronic the abuse, the higher the lifetime prevalence [27]. Subic-Wrana et al. [24] analyzed the specific relationship of trauma types and frequencies of diagnoses in a general clinical sample and found an association of emotional abuse with depression and personality disorders (see also [36]) and an association of physical abuse with somatoform disorders. However, sexual abuse was associated with adjustment and posttraumatic stress disorders [24]. Accordingly, Fernando et al. [37] reported in a study on the impact of childhood trauma on emotion regulation in borderline personality disorders and major depression significantly higher scores on emotional abuse and emotional neglect in depressed patients, yet borderline patients reported significantly more emotional abuse than did depressed patients. Bernet and Stein [38] assessed 47 adults with major depression and 41 healthy controls who were given the CTQ. Patients with major depression reported significantly more severe emotional abuse, emotional neglect, and physical abuse in childhood. Also Gibb et al. [32] found in a psychiatric outpatient study that childhood emotional abuse was related to major depression, posttraumatic stress disorder, and social phobia, whereas physical and sexual abuse were related to anxiety disorders. Etain et al. [39] confirmed this same preferential association of emotional abuse for a group of bipolar patients. And Spinhoven et al. [33] found emotional neglect as the predictor of depressive disorders, dysthymia, and social phobia.

Another central finding was the relationship between the number of different types of childhood maltreatment and the risk for mental or medical disorders (e.g., [27, 28, 30, 31, 39, 40]). Multiple traumatic experiences are also assumed to have a substantial impact on the persistence or chronicity of severe depressive symptoms (e.g., [41]). For example, Wiersma et al. [42] reported a dose-response relationship between frequencies of childhood trauma and chronicity of depression and identified childhood trauma as an independent determinant of chronicity of depression. Tanskanen et al. [28] confirmed in a large two-year follow-up community sample that life-threatening accidents, victimization by violent crime, domestic violence, and childhood sexual abuse were significantly higher among “persistent depressives” (p. 461); multiple traumatic experiences substantially increased this likelihood. Widom et al. [27] showed in their prospective longitudinal study on abused and neglected children that children who experienced multiple forms of abuse or neglect were at elevated risk for the development of a later depression. Experiences of multiple childhood trauma also correlate with severity of trauma (e.g., [43]). Khan et al. [31] confirmed this graded dose-response relationship for multiplicity of trauma and depression.

Few studies explicitly compared chronically and nonchronically depressed patients with respect to childhood trauma: Wiersma et al. [42] found besides the longer illness duration significantly more severe symptoms of depression measured by the Inventory of Depressive Symptomatology-Self-Report (IDS-SR) in chronically depressed patients against nonchronically depressed patients signaling thresholds from moderate to severe symptoms and from moderate to mild symptoms, contrastingly. Klein et al. [44] showed in a ten-year follow-up study on the course of chronic and nonchronic depression that patients with dysthymia showed slower rates of improvement over time and higher symptom levels at 10-year assessments. Further, dysthymic patients met symptom criteria for depressive disorder over a period of 60% of the follow-up, compared to 21% for patients with nonchronic depressive disorders. Hence, authors suggested chronic depression to be “more severe condition when considered from a longitudinal perspective” (p. 878).

Moreover, chronicity in contrast to nonchronicity of depression was significantly associated with emotional neglect, psychological abuse, physical abuse, and sexual abuse [42] (the ladder also more frequently in chronic compared with nonchronic outpatients [45]). Spinhoven et al. [33] reported dysthymia to show even higher significant associations with the childhood adversities emotional neglect and sexual abuse than did depression (p. 109). Riso et al. [46] elaborated in their review that the development of chronic depression compared to nonchronic forms may involve increased levels of childhood adversity. Amongst others, longer duration and higher symptom severity were each associated with multiple forms of childhood adversity in especially chronic depression [47]. A history of childhood abuse also predicted a lower probability of remission [47].

Reported studies not only were based on distinct samples but also relied on a variety of measures and different diagnostic procedures. Though several studies used the Childhood Trauma Questionnaire (CTQ) (e.g., [24, 32, 37, 38, 42, 45]), others applied childhood trauma questions (e.g., [28, 40]), scales [31], childhood trauma lists (e.g., [26]), and childhood trauma interviews (e.g., [33, 40]), or abused or neglected children were drawn from records of criminal courts (e.g., [27]). Some studies administered diagnostic interviews yielding DSM-III/IV diagnoses (e.g., [26, 31–33, 45]), and others relied on medical diagnoses (e.g., [24]) or applied symptom inventories (e.g., [27, 28, 37, 42]).

The aims of this study were to examine extent and types of retrospectively reported childhood abuse and neglect in adult patients suffering from acute chronic forms of depression operationalized as either diagnosis of dysthymia or double depression lasting for at least 24 months or longer or a major depressive episode currently lasting for at least 12 months or longer. The CTQ was used to assess patients' type and level of childhood abuses and/or neglect. We predicted that a substantially high number of the chronically depressed patients would report suffering from childhood trauma. We further assumed a substantially high number of patients reporting experiences of severe and of multiple trauma in childhood. We predicted that depressed patients with multiple trauma reports differed from patients with fewer trauma reports in symptom severity assessed by the Beck Depression Inventory (BDI-2) referring to reportedly higher symptom severity in chronically depressed patients (e.g., [42, 44, 47]). Secondly, we investigated the association between trauma types and symptom severity in chronically depressed patients and asked for the specificity of trauma types or multiplicity.

## 2. Methods

### 2.1. Participants

Chronically depressed patients (according to the criteria of the* Diagnostic and Statistical Manual of Mental Disorders*,* Fourth Edition*, DSM-IV [48]) were recruited within the German LAC Depression Study between 2007 and 2013. LAC is an abbreviation for long-term treatment of chronically depressed patients in German. The study examines the outcomes of psychotherapeutic long-term treatments and compares psychoanalytic with cognitive-behavioral therapies. The multicentre study combines a naturalistic and randomized-controlled trial design. Patients between 21 and 60 years of age were included. Patients had to currently meet a DSM-IV [48] diagnosis of either a major depressive episode already lasting for at least 12 months or longer or a dysthymia lasting for at least 24 months or longer at study intake, respectively. Hence, chronicity was operationalized as a function of time within diagnoses. In addition, patients had to meet a BDI-2 score of above 17 and a Quick Inventory of Depressive Symptoms (QIDS-C, clinician version) score of above 9. Exclusion criteria included ongoing or previous psychotic symptoms, substance dependence, dementia or decrements in cognitive functioning, a borderline, schizotypal, schizoid, paranoid, or antisocial personality disorder, a severe acute or chronic somatic disease, and acute suicidal intentions. The Ethics Review Commission of the Federal Chamber of Psychotherapy of the State of Hessen, Germany, approved the study protocol. The study was registered with ISRCTN91956346. Written informed consent was obtained from all participants after they had been provided with a full description of the study. The study design is published in detail elsewhere [49–51].

### 2.2. Measures

Diagnoses were made by independent, trained, and supervised interviewers using the German version of the Structured Clinical Interview for DSM-IV (SCID I and SCID II) [52]. Chronicity was operationalized as a function of time: Major depressive episodes had to last for at least 12 months already at study intake (which extends the diagnostic minimum requirement of two weeks). Diagnoses of dysthymia or double depression indicate depressive symptoms of at least 24 months or longer at study intake. BDI-2 [53] and the QIDS-C [54] were used to assess symptom severity. The global severity index of the Symptom-Checklist (SCL-90-R) [55] indicated self-reported global distress.

Childhood trauma was examined using the German short version of the CTQ, a 28-item, clinically and nonclinically well-validated self-report measure developed by Bernstein et al. [56–58] (for the German version see [59–61]). Only within the scale sexual abuse the perpetrator remains indefinite. The other scales capture traumatic experiences within family. Thus, the CTQ pictures traumatic experiences within primary relations actually expected to offer protection and holding (see also [24]). The retrospective self-report of childhood memories yields five subscales of neglect and abuses on a five-point Likert-scale ranging from 1 “never true” to 5 “very often true”: emotional neglect and physical neglect, emotional abuse, physical abuse, and sexual abuse. Each subscale consists of five items (for subscale details see [57, p. 2]). Higher scores signal a higher extent of traumatic experience. Emotional neglect refers to the lack of holding and support as well as the deficient provision of emotional needs such as love and belonging (item example: “*When I was growing up my family was a course of strength and support*”). Emotional abuse refers to an atmosphere of hatred, threat, demeaning, and humiliation and pictures verbal abuses on a child's sense of worth or wellbeing (item example: “*When I was growing up someone in my family hated me*”). Physical neglect refers to the lack of basic physical needs such as food and shelter and a sense of safeness (item example: “*When I was growing up I did not have enough to eat.*”) Physical abuse refers to physical assaults resulting in injury or risk (item example: “*When I was growing up I got hit so hard by someone in my family that I had to go see a doctor or go to the hospital.*”) Sexual abuse corresponds to any sexual contact including coercion between a child and an adult (item example: “*When I was growing up, someone tried to touch me in a sexual way or tried to make me touch them*”). Walker et al. [62] externally validated the measure by an experienced clinical interviewer who was blind to CTQ scores and determined whether reports of trauma histories represented clinically significant abuses or neglect. Following this clinical focus, the authors calculated cutoff scores using thresholds in order to specifically examine clinically relevant traumatic experiences. In the present study these cutoff scores were used to classify patients according to types of reported childhood neglect or abuses (see also [24, 63]). Cutoff scores according to Walker et al. [62] for each subscale (along with the ranges of scores for low, moderate, and severe trauma by Bernstein et al. [56]) were for sexual abuse ≥8 (low: 6-7; moderate: 8–12; severe: 13–25), physical abuse ≥8 (low: 8-9; moderate: 10–12; severe: 13–25), emotional abuse ≥10 (low: 9–12; moderate 13–15; severe 16–25), physical neglect ≥10 (low: 8-9; moderate 10–12; severe 13–25), and emotional neglect ≥15 (low 10–14; moderate 15–17; severe: 18–25). In order to examine the impact of multiple experiences of trauma we further considered the number of scales achieving these cutoff scores for each patient.

### 2.3. Data Analysis

Homogeneity between patient and missing data was analyzed using chi-square and Mann-Whitney tests. For further analyses missing data were excluded. The sample was first split into a group of traumatized chronically depressed patients who achieved cutoff scores according to Walker et al. [62] in the CTQ subscales, respectively, and a group of nontraumatized but still chronically depressed patients. For frequencies group differences were calculated using *χ* ^2^. Spearman correlations were used to estimate correlations between trauma subtypes. Secondly, the sample was divided into four trauma groups using an index counting the number of traumatic experiences reported: a* subthreshold trauma* group not reporting clinically relevant traumatic experiences, a* single trauma* group, reporting trauma on one scale, a* double trauma* group reporting trauma on two scales, and a* multiple trauma* group reporting trauma on three to five scales according to the above described cutoff scores, respectively. Group differences for multiplicity were tested pertaining to depression symptom severity (BDI-2) using one-way analyses of variance. Stepwise multiple regression analyses were conducted using the BDI-2 as the dependent variable and the five CTQ subscales, gender (Model 1), and multiplicity (Model 2) as independent variables. All statistical tests were two-tailed and for multiple comparisons a Bonferroni correction was applied. All computations were calculated with SPSS 22.

## 3. Results

The intention to treat sample of the LAC Depression Study included a total of 402 patients. 349 patients completed the CTQ. Missing data arose from incomplete questionnaires or patient study withdrawals. Statistical controls of homogeneity between patient and missing data using Mann-Whitney *U* tests were not significant for age at study intake (*p* = .084), BDI-2 (*p* = .176), QIDS-C (*p* = .106), and GSI/SCL-90 (*p* = .211) and were not significant using chi-square tests for gender (*p* = .868), diagnosis (*p* = .108), marital status (*p* = .825), education (*p* = .898), and number of previous outpatient treatments (*p* = .181) giving no indication for dropout distortions. The characteristics of the CTQ samples are detailed in Table 1. Age at study intake was on average 40.40 (SD 10.58) years. The female-male ratio corresponded to clinically depressed populations (male: *n* = 113; female: *n* = 236). The distribution of diagnoses is shown in Table 1. 34.7% of the patients were depressed for 24 months. The BDI-2 mean score of 32.28 and the QIDS-C at least mean score of 14.41 show severe to moderate depressive symptoms. The sample features a mean score of 1.29 (SD = 0.55) in the general severity index (GSI) of the SCL-90 indicating a fairly high psychic distress in patients. The chronicity of depression of the sample is further underlined by the high rate of 71.4% of patients who had been treated previously to study intake. 43.6% underwent more than one previous treatment.

The CTQ total score reached an average of 57.10 (SD = 18.41). In total, 75.6% of our chronically depressed patients reported clinically relevant histories of traumatic experiences in childhood on at least one scale (Table 2). 60.7% of the sample reported traumatic experiences of emotional abuse, 51.9% reported emotional neglect, and 25.2% reported sexual abuse in childhood. Means within CTQ subscale groups indicate trauma severity on thresholds from moderate to severe [56] indicating clinically relevant traumatization in childhood [62]. Gender differences were found: significantly more women reported a history of emotional abuse (*χ* ^2^ = 7.44, *p* = .006) and of sexual abuse (*χ* ^2^ = 10.83, *p* = .001). The total number of patients achieving thresholds in at least one subscale also showed significant gender differences (*χ* ^2^ = 3.97, *p* = .046).

Spearman correlations between the types of childhood trauma were fairly moderate in magnitude. The highest correlations were found between emotional abuse and emotional neglect (0.64, *p* ≤ .001). Others were lower. Subscale correlations are presented in Table 3.

In order to calculate dose-response relationships we further differentiated four trauma groups; hence an index was used counting the number of scales per patient that achieved cutoff scores for clinically relevant reports of traumatic experiences according to Walker et al. [62]:* subthreshold trauma*,* single trauma*,* double trauma*, and* multiple trauma*. The* multiple trauma* group refers to trauma reports on three to five scales. Group distribution is shown in Table 4. 37% of our patients reported multiple trauma and 18.1% double trauma. A tendency towards significant gender differences was found (*χ* ^2^ = 7.47, *p* = .058) within trauma groups. Most frequently the combination of emotional abuse and emotional neglect was reported (*n* = 37, 14.0%) followed by emotional abuse alone (*n* = 33, 12.5%). 28 (10.6%) patients achieved thresholds on all five CTQ subscales. This was followed by 9.8% (*n* = 26) achieving cutoffs for the combination of emotional abuse, emotional neglect, and physical neglect.

Table 5 shows the BDI-2 descriptive scores within the four trauma groups, respectively. The mean score increases from the single to the multiple trauma group. Interestingly, patients in the subthreshold trauma group show similar values as patients in the double trauma group. In the analysis of variance with age and gender as covariates (Table 6) we found a highly significant effect of the trauma group. Though the Levene test indicates a violation of the assumption of homogeneity of variance, results can be retained as to the large sample and the validation of this highly significant result through an additionally conducted Kruskal-Wallis test. For post hoc analyses we used the Games Howell test robust against violations of homogeneity of variance. The multiple trauma group differs significantly with higher values from the three other trauma groups.

First, a stepwise multiple regression analysis (Model 1) was performed between the BDI-2 mean score indicating symptom severity as the dependent variable and emotional, physical, and sexual abuse and emotional and physical neglect scores from the CTQ as the predictor variables (Table 7). Due to significant gender differences within the CTQ subscales, emotional abuse and sexual abuse, we included gender in the model. Results included the subscales emotional abuse and sexual abuse as explanatory variables significantly increasing the risk of symptom severity in chronically depressed patients (*R* = .251, *R* ^2^ = .063, *F* = 11.584, and *p* < .01). After emotional abuse and sexual abuse were considered in step two, the remaining abuses and neglect as well as gender failed to contribute significantly to depression symptom severity in chronically depressed patients. 

Second, a stepwise multiple regression analysis (Model 2) additionally included multiplicity operationalized as achieving cutoff scores according to Walker et al. [62] on at least three to maximum five CTQ subscales (Table 8). Results included multiplicity as remaining explanatory variable significantly increasing the risk of symptom severity in chronically depressed patients (*R* = .245, *R* ^2^ = .060, *F* = 22.071, and *p* < .01). The types of abuses and neglect as well as gender failed to contribute significantly to symptom severity in chronically depressed patients. 

## 4. Discussion

This study investigated prevalence, extent, and associations of childhood trauma exposure in a specific chronically depressed adult sample diagnosed according to DSM-IV criteria. We relied on the CTQ as an internationally widely used, reliable, and valid instrument. Chronicity was assessed through DSM diagnoses and validated by the substantial number of patients undergoing up to four treatments before study intake. This allowed comparisons between traumatized chronically depressed patients with a clinically significant history of childhood trauma and chronically depressed patients without such a history providing strength as to the possibility of differentially investigating trauma within chronically depressed patients.

Overall, the prevalence of childhood trauma was remarkably high with a magnitude of 75.6%. Moreover, CTQ subscale means indicated childhood trauma on the thresholds from moderate to severe exposure [56–58]. We additionally applied higher thresholds for the specific examination of clinically relevant childhood trauma [62]. Specific contributions of certain types of childhood trauma to the vulnerability of different forms of psychopathology were repeatedly reported. Our results show that emotional abuse was reported most frequently with 61%. Additionally, 25% of the patients reported childhood sexual abuse. Contrastingly, 15% emotional abuse and 12.6% sexual abuse were found in a representative German survey study on the prevalence of childhood trauma [64]. These findings show chronically depressed patients to be highly burdened by childhood adversities and supports reports of greater childhood adversity in chronic forms of depression compared to nonchronic forms [46]. For example, dysthymic patients were reported to have significantly poorer early parental relationships and to receive less care than patients with episodic depression [65]. Also, duration of depression was reported to be uniquely predicted by maternal abuse, maternal indifference, and paternal overcontrol [47].

In our study, women reported significantly more frequent childhood trauma in general and emotional abuse and sexual abuse in particular. McGrath et al. [66] pointed to the higher risk of victimization in women and estimated childhood abuse in women at 21.7 to 37%. Lampe's [67] review on childhood trauma confirmed that women suffered more frequently from sexual abuse than men. Scher et al. [63] showed that women were nearly twice as likely to report emotional abuse and four times as likely to report sexual abuse. Furthermore, our results showed that when referring to associations of trauma types and trauma groups with symptom severity, gender did not contribute significantly. Arnow et al. [68] examined the moderating role of gender on the association between childhood abuse, neglect, and depression, yet they found no gender differences. However, they also identified significantly more depressed women than men reporting histories of emotional abuse and sexual abuse, which they interpreted as stemming from higher rates of victimization among women (p. 179). The lack of evidence for gender differences in the relationship of childhood adversity and depression was also substantiated by other studies reporting that among those with a history of childhood sexual abuse [35] with physical [69] or emotional abuse [69, 70] men and women were equally at risk for depression.

Mainly, our results suggest a substantial influence of multiple childhood trauma on a severe and chronic course of depression in adulthood. 37% of our patients reported a history of multiple childhood trauma, that is, trauma reports on at least three to maximum five CTQ scales. Additionally, 18.1% achieved thresholds on at least two subscales. Also in line with other studies [27, 28, 40, 42], the patients reporting multiple childhood trauma showed greater symptom severity suggesting a dose-response relationship between the number of childhood maltreatments and symptomatology. This suggests that cooccurrences of childhood trauma and its possible effects being synergistic or additive [12, 71] due to cumulative, sequential, simultaneous, and/or complex [16, 17] impacts may be specifically relevant in chronically depressed patients. This “cumulative effect of chronic exposure to multiple adversities” (p. 95) [72] was in general stressed as the largest burden of diseases within the ACE study.

Moreover, results indicated that significant associations between the trauma types, emotional abuse and sexual abuse, and symptom severity (Model 1) in chronically depressed patients were not maintained when taking multiplicity (Model 2) into account. Though both regression models only accounted for a poor amount of variance (4–6%), results replicated empirical findings (see, e.g., a meta-analysis on nonsexual maltreatment [43] reporting robust evidence for the relationship of emotional abuse and depression or a systematic review on the evidence of the relationship between childhood sexual abuse and mental disorders reporting odds ratios of 1.1 (p. 1886) for depression [73]). Yet, these studies did not take multiplicity of childhood trauma exposures into account. Multiplicity was also related to severity [30, 43, 74] which we supported referring to our figures of CTQ subscale distributions and mean values.

Fischer and Riedesser [12] stress childhood sexual abuse as a serious relational trauma and conceptualized childhood sexual abuse itself as multiple trauma (p. 303). Wetzel [75], for example, found that 64.3% of sexually abused participants were at the same time physically abused. Molnar et al. [26] examined the relation between childhood sexual abuse and later psychopathology and reported significantly higher percentages of women and men with lifetime dysthymia (15.7% and 12.5%) and depression (39.3% and 30.3%) among those reporting childhood sexual abuse. Briere and Elliott [76] linked experiences of childhood sexual abuse to disruptions in the development of a sense of self, causing difficulties in relating to others. Unfortunately the CTQ subscale for sexual abuse lacks information on perpetrators and the victims' relations to them making conclusions on familial versus nonfamilial sexual abuse impossible.

With emotional abuse depression is again associated with trauma as a “relational” term. This type captures a narcissistic dimension of hatred, threat, demeaning, and humiliation and pictures verbal abuses on a child's sense of worth or wellbeing also embodying experiences of repeated losses in primary relations. Emotionally abused children may also attribute more negatively than nonabused children which may lead to the development of a general negative attribution style [77–80]. For example, Gibb and Abela [81] investigated the potential of emotional abuse to change children's inferential styles in a sample of 140 children of parents with a depression history. They found that emotional abuse was significantly related to changes in children's inferential styles regarding consequences and self-characteristics.

Beyond multiplicity, Khan et al. [31] argued for specific sensitive exposure periods exerting maximal impact on risk for major depression (see p. 23). The authors caution that the linear increase associated with exposure to the number of different types of maltreatment may be a statistical byproduct of the fact that exposure to more types of abuse increases the chance of experiencing a critical form of maltreatment at a critical age. Their results showed that being rejected at the age of 14 was a crucial risk for depression: females were particularly sensitive to peer emotional abuse and males to parental nonverbal emotional abuse at the age of 14, respectively. Unfortunately, the CTQ neither allows an investigation of critical developmental periods (p. 23) nor asks for peer abuse. Rather, the CTQ captures an atmosphere of primary relations and trauma comprised of a loss of sense of self-worth, wellbeing, or self-efficacy (e.g., [8, 9, 82, 83]). This allows linkages to psychoanalytical conceptions of inner objects through processes of identification and introjection (e.g., [9, 82]) or to attachment theory (e.g., [84–86]) that might explain the strong connection of multiple trauma exposure to symptom severity in chronically depressed patients. It marks the disruption of one's capacity to ascribe meaning to trauma, the loss of a basic sense of trust, and an enduring shock of one's understanding of the outer and the inner world [13, 14, 83]. Fonagy et al. [87] showed that rates of insecure attachment in dysthymia were significantly higher than in major depression.

Strengths of this study include a large sample of patients solely diagnosed with chronic forms of depression using structured diagnostic interviews and the use of a well-validated instrument of childhood abuse. However, this study design also has several limitations: the retrospective assessment of childhood abuse and neglect may be subject to recall biases. Yet, adults' recall on trauma is reported to be relatively reliable [88, 89]. Along with good criterion-related validity with therapist's ratings of abuse [58], we also applied thresholds specifically developed to investigate clinically significant histories of childhood trauma [62]. Nevertheless, the measure captures specific types of trauma in contrast, for example, to early loss of parents, natural catastrophes, physical or mental disease in childhood or of parents, and peer abuses. Future research would especially benefit from focusing on a longitudinal aspect that allows a direct examination of trauma exposure and its relation to chronic depression and therapy outcomes.

Our findings suggest implications for clinical practice. Clinicians should precisely look for the presence of childhood trauma in chronically depressed patients being aware of its possible prognostic implications. In particular multiple traumatic experiences, emotional abuse, and sexual abuse may lead to a more chronic and severe course of depression. Nonetheless, a group of likewise severely depressed patients reporting histories of childhood trauma subthreshold as conceptualized within this study remained. This underlines that childhood trauma may only be one central pathway leading to chronic depression in adulthood (e.g., [8, 9, 12, 13, 19–21, 89, 90]). Depression summarizes a group of highly complex mental disorders that have to be diagnosed precisely and treated cautiously in consideration of its origins. For example, patients with a history of childhood trauma showed a poorer medication response than those without such a history and were significantly more likely to remit with psychotherapy than pharmacotherapy [47].

## 5. Conclusion

Consistent with previous studies showing an association of childhood trauma and depression, in our study a high number of chronically depressed patients experienced not only childhood trauma, but also multiple trauma. The magnitude in frequency of traumatized depressives and of multiply traumatized depressed patients remarkably stresses the impact of trauma within chronic courses of depressive disorders and the necessity to intensively and critically integrate this trauma aspect in treatments. Particularly multiple childhood trauma might be specifically related to chronic courses of depression.

## Figures and Tables

**Table 1:** Demographic and clinical characteristics of the sample (*n* = 349).

	Mean	SD
Age	40.40	10.58
CTQ total score	57.10	18.41
BDI-2	32.28	8.17
QIDS-C	14.41	3.16
SCL-90 (GSI)	1.29	0.55

	*N*	%

Gender		
Female	236	67.6
Male	113	32.4
Nationality		
German	307	88
Other than German	42	22
Education		
9 years	27	7.7
10 years	84	24.1
12 years	228	65.3
Other	7	2.1
Marital status		
Single	199	57.0
Married	87	25.0
Separated/divorced	59	17.1
Widowed	1	0.3
Other	3	0.9
Diagnosis		
Major depressive episode	228	65.3
Double depression	81	23.2
Dysthymia	40	11.5
Number of previous outpatient treatments		
None	91	26.1
One	97	27.8
Two and three	112	32.1
Four and more	40	11.5
Missing	9	2.6

**Table 2:** Frequencies, means, and standard deviations of depressed patients achieving cutoff scores in CTQ subscales (*N* = 349).

CTQ scales	*N*	%	Male	%	Women	%	Mean	SD
Total number^1^	264	75.6	78	69.0	186	78.8	63.50	16.49
Emotional abuse	212	60.7	57	50.4	155	65.7	15.47	4.23
Emotional neglect	181	51.9	57	50.4	124	52.5	18.86	2.86
Physical neglect	111	31.8	30	26.5	81	34.3	12.01	2.23
Physical abuse	94	26.9	27	23.9	67	28.4	11.32	3.80
Sexual abuse	88	25.2	16	14.2	72	30.5	12.20	4.49

^1^Achieving cutoff score in at least one scale.

**Table 3:** Spearman correlations among the five CTQ subscales (*N* = 349).

	Emotional neglect	Physical neglect	Physical abuse	Sexual abuse	BDI-2
Emotional abuse	.64^*∗∗*^	.48^*∗∗*^	.51^*∗∗*^	.55^*∗∗*^	.22^*∗∗*^
Emotional neglect		.63^*∗∗*^	.40^*∗∗*^	.36^*∗∗*^	.16^*∗∗*^
Physical neglect			.40^*∗∗*^	.38^*∗∗*^	.15^*∗∗*^
Physical abuse				.45^*∗∗*^	.17^*∗∗*^
Sexual abuse					.21^*∗∗*^

^*∗∗*^ *p* ≤ .001.

**Table 4:** Four trauma groups: subthreshold, single, double, and multiple trauma.

Trauma groups	*N*	%	Male	%	Female	%
Subthreshold trauma	85	24.4	35	41.2	50	58.8
Single trauma	72	20.6	25	34.7	47	65.3
Double trauma	63	18.1	22	34.9	41	65.1
Multiple trauma	129	37.0	31	24.0	98	76.0
Total	349	100	113	32.4	236	67.6

**Table 5:** Descriptive scores  for BDI-2 and trauma groups.

Measures	Subthreshold trauma (*N* = 85)		Single trauma (*N* = 72)		Double trauma (*N* = 63)		Multiple trauma (*N* = 129)	
	M	SD	M	SD	M	SD	M	SD
BDI-2	31.29	8.14	29.51	6.31	31.43	7.69	34.88	8.67

**Table 6:** Analysis of variance (ANOVA) according to trauma group conducted for BDI-2 and age and gender as covariates^1^.

Factors	df	*F*	*p*	
Gender	1	1.62	.205	

Age	1	0.33	.569	

Trauma group	3	7.18	.000	Post hoc^2^ 1 < 4 *p* = .013 2 < 4 *p* = .000 3 < 4 *p* = .029

^1^Levene *F*-test *p* = .032, additional Kruskal-Wallis *H*-test: chi^2^ = 21.00, *p* = .000.

^2^Post hoc test: Games Howell.

**Table 7:** Results of stepwise regression analysis for depression symptom severity (BDI-2), trauma subtypes, and gender (*n* = 349) (Model 1).

Variables	*B*	SE *B*	*β*	*T*
*Step 1*				
Emotional abuse	.335	.080	.219^*∗∗*^	4.184
				
Physical abuse	.082		.072	
Sexual abuse	.135		.124^*∗*^	
Emotional neglect	.038		.030	
Physical neglect	.059		.052	
Gender	.065		.066	
*R*	.219^*∗∗*^			
*R* ^2^	.048			
Adj. *R* ^2^	.045			

*Step 2*				
Emotional abuse	.244	.089	.159^*∗∗*^	2.744
Sexual abuse	.294	.126	.135^*∗*^	2.332
				
Physical abuse	.049		.042	
Emotional neglect	.045		.036	
Physical neglect	.037		.033	
Gender	.053		.066	
*R*	.251^*∗*^			
*R* ^2^	.063			
Adj. *R* ^2^	.057			
Mean BDI-2	32.28			

^*∗*^ *p* ≤ .05.

^*∗∗*^ *p* ≤ .001.

**Table 8:** Results of stepwise regression analysis for depression symptom severity (BDI-2), trauma subtypes, gender, and multiplicity (*n* = 349) (Model 2).

Variables	*B*	SE *B*	β	*T*
*Step 1*				
Multiplicity	1.378	.293	.245^*∗∗*^	4.698
				
Emotional abuse	.107		.085	
Physical abuse	.051		.421	
Sexual abuse	.114		.103	
Emotional neglect	.025		.020	
Physical neglect	.011		.009	
Gender	.064		.066	
*R*	.245^*∗∗*^			
*R* ^2^	.060			
Adj. *R* ^2^	.057			
Mean BDI-2	32.28			

^*∗∗*^ *p* ≤ .001.
